# Fluid management of the neurological patient: a concise review

**DOI:** 10.1186/s13054-016-1309-2

**Published:** 2016-05-31

**Authors:** Mathieu van der Jagt

**Affiliations:** Department of Intensive Care (Office H-611) and Erasmus MC Stroke Center, Erasmus Medical Center Rotterdam, P.O. Box 2040, 3000 CA Rotterdam, The Netherlands

**Keywords:** Subarachnoid haemorrhage, Traumatic brain injury, Fluid management, Volume status, Hypervolemia, Haemodynamic monitoring

## Abstract

Maintenance fluids in critically ill brain-injured patients are part of routine critical care. Both the amounts of fluid volumes infused and the type and tonicity of maintenance fluids are relevant in understanding the impact of fluids on the pathophysiology of secondary brain injuries in these patients. In this narrative review, current evidence on routine fluid management of critically ill brain-injured patients and use of haemodynamic monitoring is summarized. Pertinent guidelines and consensus statements on fluid management for brain-injured patients are highlighted. In general, existing guidelines indicate that fluid management in these neurocritical care patients should be targeted at euvolemia using isotonic fluids. A critical appraisal is made of the available literature regarding the appropriate amount of fluids, haemodynamic monitoring and which types of fluids should be administered or avoided and a practical approach to fluid management is elaborated. Although hypovolemia is bound to contribute to secondary brain injury, some more recent data have emerged indicating the potential risks of fluid overload. However, it is acknowledged that many factors govern the relationship between fluid management and cerebral blood flow and oxygenation and more research seems warranted to optimise fluid management and improve outcomes.

**Electronic supplementary material:**

The online version of this article (doi:10.1186/s13054-016-1309-2) contains supplementary material, which is available to authorized users.

## Background

Fluid management in critically ill brain-injured patients is aimed at maintaining adequate cerebral blood flow (CBF) and oxygenation. However, fluid management in brain-injured patients has several distinctive features compared with non-brain-injured critically ill patients: (1) fluid tonicity is a more pertinent issue; (2) tissue oedema not only results in oxygen diffusion impairments but may also impair CBF due to the unfavourable volume–pressure characteristics of the intracranial content; (3) fluid management is commonly regarded as ‘basic care’ in brain injury, whereas fluid management in other critically ill patients is commonly guided by haemodynamic monitoring, rendering it ‘intensive care’; and (4) optimising CBF with adequate fluid management seems intrinsically more challenging than systemic circulation, because sophisticated monitoring tools for CBF and cerebral oxygenation are generally less well implemented in clinical practice. These distinctive features of fluid management in brain-injured patients deserve scrutiny, because recent data (both within and outside the area of neurocritical care) suggest that the ‘basic care’ of fluid administration in brain-injured patients may have an impact on outcome [1–3]. This is especially salient because fluid management practices in brain-injured patients are highly variable [4, 5], which may partly be caused by the fact that published guideline recommendations on fluid management [6, 7] are based on low-grade evidence or may be perceived as imprecise (e.g. ‘euvolemia’ is subject to interpretation).

The aim of this narrative review is: to summarize existing guidelines and contemporary literature on routine (maintenance) fluid management in critically ill brain-injured patients (traumatic brain injury (TBI), subarachnoid haemorrhage (SAH), intracerebral haemorrhage (ICH), ischaemic stroke), with a focus on the amounts and types of fluids and volume and circulatory status monitoring; and to discuss practical issues of fluid management.

## Pathophysiological considerations

**Fig. 1:**
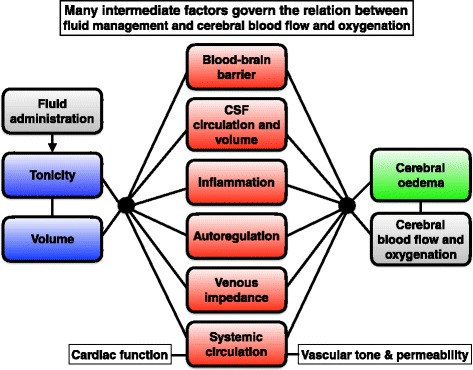
The effect of fluid management on CBF and cerebral oxygenation is complex because many intermediate variables exist that should be taken into account to fully appreciate possible cause and effect relationships. Some concepts relating to such intermediate variables are succinctly reviewed in the main text. *CBF* cerebral blood flow, *CSF* cerebrospinal fluid

Some basic concepts are relevant to understand effective fluid management in brain injury. The influence of fluid administration or volume status on CBF and cerebral oxygenation is complex because many factors determine the influence of the first on the latter (Fig. 1). In addition, critically ill brain-injured patients are particularly prone to disturbances of intravascular volume, electrolyte and osmotic disturbances due to central neuroendocrine disturbances and use of therapies that perturb water and sodium homeostasis, further complicating effective fluid management.

### Tonicity

Osmolality of plasma and brain interstitial fluid and CSF are equal under normal circumstances [8]. Hypotonic fluids cause water shifts to the brain because the blood–brain barrier (BBB) is water permeable whereas hypertonic fluids are well known for their ability to cause brain dehydration, both when the BBB is intact and is disrupted [9, 10]. Neurons can compensate for such fluid shifts by active solute depletion to the extracellular compartment to cause reactive ‘shrinkage’, and the BBB endothelial and other highly specialized cells within the so-called neurovascular unit will operate similarly to expel water to the intravascular compartment [11]. However, BBB disruption locally abolishes its ability to control homeostasis of electrolytes, water and other solutes, and fluid shifts will become more dependent on local pressure differences between the intravascular and extravascular compartment than osmotic tension. In contrast to peripheral tissues, where endothelium is highly permeable to electrolytes and oedema formation is more or less proportional to the infused volume of isotonic fluids, electrolytes do not distribute freely through an intact BBB. This is a key mechanism protecting the brain from oedema even when very high amounts of isotonic fluids are administered [11].

### Oedema

Cerebral oedema is stratified depending on location (intracellular or extracellular) and BBB disruption. Cytotoxic oedema is the cellular oedema of neurons or astrocytes and is the result of mainly sodium and water shifts into the cells after an insult with ATP depletion and mitochondrial dysfunction [8, 12]. Vasogenic oedema represents both water and albumin shifts through disrupted endothelial tight junctions. An intermediate type of oedema is ionic oedema, resulting from compensatory solute and water shifts from the vascular compartment to the interstitium through an intact BBB after the formation of cytotoxic oedema has decreased interstitial osmolality.

### Autoregulation

Autoregulation concerns the capacity of the blood vessels in the brain to sustain CBF by vasodilation or vasoconstriction over a wide range of systemic blood pressures, and in a more general sense may be regarded as the capacity of brain vessels to regulate blood flow in response to changes in metabolic needs. The connection between volume status and intact autoregulation relates to increased CBF to preserve oxygen delivery in response to fluid loading and decreased haematocrit or to maintaining constant CBF through vasodilation when blood pressure drops due to hypovolemia.

### Venous outflow impedance

Perfusion pressure determinants are both upstream and downstream pressures, with upstream pressures being arterial and downstream pressures being venous. Both lower arterial pressures and higher venous pressures will theoretically result in lower perfusion pressures, albeit with different consequences (i.e. low flow versus tissue oedema) [13]. Increased central venous pressure (CVP) may impede venous outflow from the brain and contribute to increased intracranial pressure (ICP) or cerebral oedema. However, increased CVP will in principle not be transferred to the intracranial compartment so long as intracranial venous structures are collapsed under the influence of ICP before exiting the cranium, and ICP cannot be affected by the extracranial CVP that is generally much lower than the ICP (waterfall effect) [14]. Consequently, venous pressure transferral back to the intracranial contents is possible when ICP is low compared with either CVP or positive-end expiratory pressure (PEEP) in mechanically ventilated patients with brain trauma [15, 16], or when several adverse circumstances act simultaneously to antagonize brain compliance (e.g. hypotonic fluid loading, high CVD, recent brain injury with oedema) as has been shown in animal experiments, but investigations have yielded contradictory results [17, 18]. Although high PEEP may influence ICP on the ‘venous side’ via pressure back-transferral, it may also and independently influence ICP on the ‘arterial side’ depending on whether autoregulation is intact (e.g. when intact, PEEP impedes venous return, resulting in arterial hypotension with cerebral vasodilation and ICP surges) [16].

## Overview of literature

### Guidelines

Contemporary recommendations for routine fluid and intravascular volume management are available from several guidelines and consensus conferences [6, 7, 19–21]. The 2007 Brain Trauma Foundation guidelines [22] do not provide specific recommendations on fluid management reflecting the pressure-oriented approach. The guideline and consensus recommendations are presented in Table 1. In SAH, euvolemia is recommended to prevent delayed cerebral ischaemia (DCI), routine hypervolemia is not recommended and hypotonic fluids and volume contraction are to be avoided. Furthermore, haemodynamic monitoring to guide fluid management is not advised routinely. Vigilant fluid balance assessment is advised to guide fluid administration but aggressive fluid administration aimed at hypervolemia is considered harmful. The consensus statement on multimodality monitoring in neurocritical care [19] recommends haemodynamic monitoring in patients with haemodynamic instability. Guidelines on ischaemic stroke highlight the importance of isotonic rather than hypotonic fluids and avoidance of hypovolemia and dextrose solutions [20, 21].

**Table 1:** Summary of guideline/consensus conference recommendations on routine fluid and circulatory volume management in brain-injured patients

	Recommendations on routine fluid management and volume status	
Source	Monitoring	Management
AHA/ASA SAH guidelines (2012) [7]	1. Monitoring volume status in certain patients with recent aneurysmal SAH by some combination of central venous pressure, pulmonary wedge pressure and fluid balance is reasonable, as is treatment of volume contraction with crystalloid or colloid fluids. (Class IIa, evidence level B)	1. Maintenance of euvolemia and normal circulating blood volume is recommended to prevent DCI. (Class I, evidence level B) 2. Prophylactic hypervolemia […] before the development of angiographic spasms is not recommended. (Class III, evidence level B) 3. Administration of large volumes of hypotonic fluids and intravascular volume contraction is not recommended. (Class III, evidence level B)
Neurocritical Care Society recommendations on critical care management in SAH (2011) [6]	1. Monitoring of volume status may be beneficial. (Moderate quality evidence; weak recommendation) 2. Vigilant fluid balance management should be the foundation for monitoring intravascular volume status. While both non-invasive and invasive monitoring technologies are available, no specific modality can be recommended over clinical assessment. (Moderate quality evidence; weak recommendation) 3. Central venous lines should not be placed solely to obtain CVP measures and fluid management based solely on CVP measurements is not recommended. (Moderate quality evidence; strong recommendation) 4. Use of PACs incurs risk and lacks evidence of benefit. Routine use of PACs is not recommended. (Moderate quality evidence; strong recommendation)	1. Intravascular volume management should target euvolemia and avoid prophylactic hypervolemic therapy. In contrast, there is evidence for harm from aggressive administration of fluid aimed at achieving hypervolemia. (High quality evidence; strong recommendation) 2. Isotonic crystalloid is the preferred agent for volume replacement. (Moderate quality evidence; weak recommendation) 3. In patients with a persistent negative fluid balance, use of fludrocortisone or hydrocortisone may be considered. (Moderate quality evidence; weak recommendation)
Consensus statement on multi-modality monitoring in neurocritical care (2014) [19]	1. We recommend that hemodynamic monitoring be used to establish goals that take into account cerebral blood flow (CBF) and oxygenation. These goals vary depending on diagnosis and disease stage. (Strong recommendation, moderate quality of evidence) 2. We recommend the use of additional haemodynamic monitoring (e.g. intravascular volume assessment, echocardiography, cardiac output monitors) in selected patients with haemodynamic instability. (Strong recommendation, moderate quality of evidence) 3. We suggest that the choice of technique for assessing pre-load, after-load, cardiac output and global systemic perfusion should be guided by specific evidence and local expertise. (Weak recommendation, moderate quality of evidence)	Not applicable
Brain Trauma Foundation guidelines on traumatic brain injury (2007) [22]	No recommendations	No recommendations
AHA/ASA guidelines for the early management of patients with acute ischaemic stroke (2013) [20]	No recommendations	1. Daily fluid maintenance for adults estimated as 30 ml/kg body weight 2. Use isotonic fluids rather than hypotonic fluids (might exacerbate ischaemic brain oedema) 3. Hypovolemia should be corrected with i.v. normal saline
AHA/ASA Recommendations for the management of cerebral and cerebellar infarction with swelling [21]	No recommendations	1. Use of adequate fluid administration with isotonic fluids might be considered. (Class IIb, evidence level C) 2. Hypotonic or hypo-osmolar fluids are not recommended. (Class III, evidence level C)

*AHA/ASA* American Heart Association/American Stroke Association, *CVP* central venous pressure, *DCI* delayed cerebral ischemia, *PAC* pulmonary artery catheter, *SAH* subarachnoid haemorrhage

### Maintenance fluids: how much?

The current guidelines on fluid management in brain injury recommend using fluid balances to guide volume status (Table 1). A non-systematic overview of pertinent contemporary studies in brain-injured patients is provided in Additional file 1 [3, 23–45]. Not all of the reports in this overview studied fluid balance or fluid intake as the primary aim, but because fluid amounts were clearly reported some relevant information could be extracted.

The mean fluid intake was around 3–4 L/day in SAH patients who were treated with normovolemia or received fluid management based on volumetric haemodynamic monitoring versus 4–5 L/day in patients managed with hypervolemic treatment which often included CVP or pulmonary artery occlusion pressure (PAOP)-directed management. Fluid balances generally did not differ between both treatment groups and varied around neutral balance (−0.5 to +1 L) even in a study where mean daily fluid intake was >8 L [28]. Only one study [30] included weight-normalized fluid intake (ml/kg/day). Positive fluid balances have been associated with (angiographic) vasospasm, longer hospital length of stay and poor functional outcomes [27, 37] (see Additional file 1). Higher fluid intake has been associated with more cardiovascular side effects and DCI/delayed ischaemic neurologic deficit (DIND)/infarctions [25, 27, 28, 30, 31, 34, 35]. One may argue that the adverse prognostic value of aggressive fluid loading may reflect more intense treatments in more severely affected patients rather than causal associations because many of these studies are observational cohort studies undoubtedly prone to confounding.

In the trial on prophylactic hypervolemia after aneurysm clipping after SAH by Lennihan et al. [46] the hypervolemic group had a mean fluid intake of up to 4.5 L/day versus around 3.7 L/day in the normovolemia group, with similar daily net fluid balances in both groups (between +0.7 and −0.7 L/day). Hypervolemia did not confer any benefit with regard to CBF or clinical outcomes. The trial by Egge et al. [47] randomized SAH patients between prophylactic hypertensive hypervolemic haemodilution (triple-H) and normovolemia, and reported fluid intake of approximately 3 L/day in the normovolemic group versus 4–5 L/day in the triple-H group (no exact data were provided in the publication). There were no differences in clinical endpoints, but more complications with triple-H (extradural haematoma, haemorrhagic diathesis, congestive heart failure and arrhythmia). For fluid balances (in contrast to fluid intake) such a trend for DCI/DIND/vasospasm was less clear, although two studies reported more adverse outcomes (not restricted to DCI) associated with positive versus negative fluid balances. Data from three other RCTs (of which two were by the same group) [25, 34, 35], a propensity matched analysis on prospective data from a RCT in SAH patients [31] and a RCT on echocardiography-guided fluid resuscitation in trauma patients [43] corroborated the association between more aggressive fluid loading and adverse outcomes (DCI/DIND, cardiovascular side effects, pulmonary oedema, functional outcome and mortality) in both SAH and TBI patients. In addition, a population-based study (*n* = 5400) reported a temporal association between increased fluid intake and mortality when administered in the pre-DCI period in SAH patients (days 1–3 after the bleed), although it seemed to be beneficial in the DCI risk period (days 4–14) [30]. The data from the RCTs, the propensity matched analysis and the population-based study suggest that there may indeed be a causal link between aggressive fluid loading beyond euvolemia and adverse neurological outcomes, since major confounding is much less likely in these studies. However, tailoring treatment in individual patients remains important, which is exemplified by an investigation in SAH patients showing that increased fluid intake was associated with DIND whereas net negative fluid balances seemed harmful, but only in patients with severe vasospasm [31]. In line with this study and the fact that frank hypovolemia is to be avoided in brain-injured patients, a study in TBI patients found an association of negative fluid balances (< −594 ml) with poor outcome [42]. The ICP and CPP values did not differ between outcome groups, which may indicate that fluid management might impact on outcomes despite successful pressure-targeted management in TBI [42]. Studies showing harm from more positive fluid balances and higher fluid intake and studies specifically targeting fluid management with isotonic fluids are scarce in TBI compared with SAH [42, 45, 48].

### Maintenance fluids: which ones?

A recent review summarized current knowledge on risks and benefits of different types of fluids that are used in traumatic brain injuries [49], and therefore this will not be dealt with in depth here. Some key points regarding the fluid compounds in brain-injured patients are as follows: (1) isotonic fluids are the mainstay of maintenance fluid therapy [50]; (2) synthetic colloids may be harmful after SAH [31, 51] and have not been thoroughly investigated in TBI; (3) contrasting evidence on albumin exists in TBI—its use has been associated with both harm (SAFE study [52]) and benefit [53], but consensus exists that it should generally not be used in TBI and in SAH there is currently insufficient evidence on definite benefit from albumin [54]; (4) in SAH, standard fluid management with saline may have alternatives with more balanced solutions resulting in more stabile electrolytes, less fluid intake and less activation of the pituitary axis stress hormones (cortisol, TSH) [55]; and (5) sodium lactate may hold promise as an alternative fluid to saline solutions in routine fluid management in severe TBI because a recent pilot RCT showed improved ICP control, better electrolyte profile and decreased fluid intake, and its use may have interesting metabolic benefits for the injured brain and its susceptibility to secondary injuries [40]. Of note with regard to the SAFE study, equipoise exists regarding whether the adverse effects of albumin on ICP were related to the relative hypotonicity of the 5 % solution or leakage of albumin through a disrupted BBB creating oncotic shifts that promote oedema [56].

### Monitoring of volume and circulatory status

A comprehensive literature search by delegates from a 2010 SAH consensus conference that selected studies on clinical monitoring and volume status (*n* = 16) highlighted several important findings [57]. First, bedside assessment of volume status is not accurate because sensitivity and positive predictive values for hypovolemia and hypervolemia were less than or equal to 0.37 and 0.06 respectively. These data seem to call into question the effectiveness of vigilant fluid balance management in establishing euvolemia. Second, blood volume measurements to guide fluid management seem feasible and may contribute to the prevention of hypovolemia, but these results are from a small study and blood volume measurements are not widely available. Third, transpulmonary thermodilution (TPT) techniques seem feasible to guide fluid management after SAH. The concluding remarks of this literature search focused on fluid ‘imbalance’, but stressed hypovolemia as a more stringent problem after SAH than hypervolemia. A recent systematic review on advanced hemodynamic monitoring in brain-injured patients (SAH, cardiac arrest, TBI, stroke [58]) showed that such monitoring is widely applied using many different protocols based on local experience. Many other—sometime contradictory—associations between haemodynamic parameters and clinically relevant outcomes were found, but the authors concluded that more research is necessary. The publication showed that the relation between systemic haemodynamics and cerebral perfusion and oxygenation was scarcely studied [58].

#### Transpulmonary thermodilution

In SAH patients, TPT monitoring seems a feasible method of assessing volume status and may help to improve outcome [23, 25, 34]. SAH patients had lower global end-diastolic index (GEDI, as a parameter for cardiac preload) but higher cardiac index immediately after SAH, related to increased catecholamines indicating sympathetic activation. The increased cardiac output in spite of reduced GEDI is difficult to explain by true hypovolemia, since this would result in low GEDI and low cardiac output. Splanchnic vasoconstriction with acute fluid shifts from the abdominal to the thoracic compartment was described in animal experiments as a causal mechanism for neurogenic pulmonary oedema in acute brain trauma [59], and may explain volume contraction in the situation of increased cardiac output [60]. A relation between lower GEDI and the occurrence of DCI has been described but whether this reflects true hypovolemia remains to be established [33]. With TPT, fluid intake could be significantly reduced as compared with a fluid strategy aiming at a CVP of 5–8 mmHg, resulting in less DCI and a trend towards better functional outcome [25], confirmed in a subsequent study by the same investigators [34]. Another study found that influencing GEDI and cardiac output by ‘triple-H’ did not succeed despite effectively higher fluid intake and blood pressures [32].

#### Fluid responsiveness

Fluid responsiveness (increased cardiac output in response to a fluid challenge) in patients with cardiac output monitoring may help to improve cerebral oxygenation (partial pressure of brain tissue oxygen (PBrO_2_)), which was indeed nicely shown in a recent study in SAH patients: fluid responsiveness was associated with improvements in PBrO_2_ and cerebral perfusion pressure [61]. In contrast, other studies in both SAH and TBI patients [62, 63] could not confirm such associations between fluid loading or cardiac output and CBF or PBrO_2_. Intravascular pressures, especially CVP, have not been shown to be particularly useful as clinical parameters to assess fluid responsiveness [64]. In contrast, vena cava distensibility is described as a reliable dynamic indicator of volume status in SAH patients and may hold promise for clinical use [65].

## Fluid management in critically ill brain-injured patients: practical issues

### Goals of fluid management

In line with the consensus statement on multimodality monitoring in neurocritical care (Table 1 [19]) the goal of fluid management is optimization of cerebral perfusion and oxygenation and minimizing secondary brain insults. Importantly, adequate fluid management in brain injury should preferably be guided by some measure of brain function as a reflection of adequacy of cerebral perfusion and oxygenation, since these are the actual endpoints of fluid titration.

### Volume status: how to define in brain injury?

There is broad consensus that hypovolemia should generally be avoided in acute brain injuries. Hypovolemia in this context may be defined as an intravascular volume that is insufficient to sustain minimally adequate cerebral perfusion and oxygenation. Euvolemia may be defined as an intravascular volume that sustains the required cerebral perfusion for adequate brain oxygenation. Defining ‘hypervolemia’ in brain injury is less straightforward. Of note, the distinctive feature of hypervolemia versus hypovolemia or euvolemia is the fact that it concerns what is outside the circulation (i.e. the extravascular space), which makes its assessment and definition much more difficult. For comparison, clinical examples outside neurocritical care are oliguria in fluid-overloaded septic and decompensated heart failure patients representing venous congestion [66]. Obviously, these situations with oliguria do not require fluid loading, since venous congestion will then increase and the ‘congestive kidney failure’ worsen. An increase in CVP will promote tissue oedema, resulting in a dilution of the capillaries and increased tissue diffusion distances for oxygen to the cells. This definition of hypervolemia derived from systemic circulation conflicts with the general use of ‘hypervolemia’ within the older SAH literature, since this designation has been associated with potential benefit for ‘clinical vasospasm’ (DCI) in some classic studies that assumed beneficial effects of ‘hypervolemia’ on blood rheology and prevention of hypovolemia [67, 68]. Further, because definitions of ‘hypervolemia’ as a therapeutic strategy have not been uniform in previous studies, comparability of these studies is hampered [69].

### A practical approach to fluid management; example for SAH

A practical approach to fluid management in brain-injured patients may include: maintenance fluid volumes routinely administered, the type(s) of fluids allowed and their tonicity; and triggers for more advanced haemodynamic monitoring. Monitoring may include invasive methods (e.g. TPT-guided) or less invasive methods (e.g. oesophageal Doppler) [65]. Further, fluid management based on fluid responsiveness [70], other dynamic hemodynamic measures (e.g. pulse pressure variation) or volumetric measures of preload (e.g. GEDI) [25] may be favoured over filling-pressure measures such as PAOP [71].

**Fig. 2:**
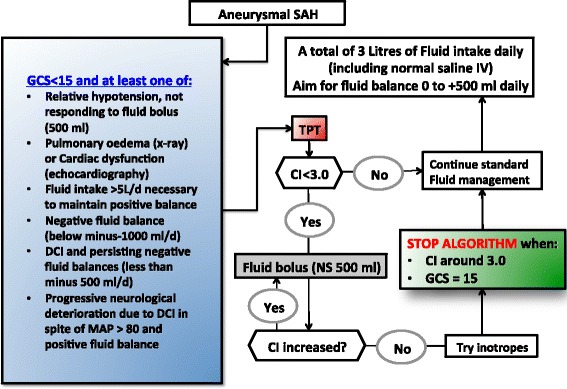
Fluid management algorithm as applied in the author’s institution in critically ill SAH patients. Principles underlying the algorithm include: define maintenance fluids (40 ml/kg/day); use isotonic crystalloid fluids; define triggers for more advanced haemodynamic monitoring and define haemodynamic goals, titrate management to these goals and give stopping rules to abort algorithm after improvements. In a subset of high-risk SAH patients, this algorithm resulted in significant reductions in fluid intake whilst maintaining cardiac output and preload indices, thus avoiding hypovolemia [75], in line with a previous study [25]. Both dynamic (e.g. fluid responsiveness) and static (e.g. GEDI) measures of volume status may thus be used to guide fluid administration. *SAH* subarachnoid haemorrhage, *TPT* transpulmonary thermodilution-based haemodynamic monitoring, *DCI* delayed cerebral ischaemia, *MAP* mean arterial pressure, *NS* normal saline (0.9 %), *CI* cardiac index (L/min/m^2^), *GCS* Glasgow Coma Scale

An algorithm has been used with success by the author in critically ill SAH patients to significantly reduce fluid intake whilst maintaining sufficient cardiac output and indices of cardiac preload (Fig. 2). This algorithm serves as an example of how the basic tenets already described may be materialized and made practical. Maintenance fluids should generally be aimed at 30–40 ml/kg/day of isotonic crystalloids (normal saline 0.9 %), with SAH patients generally needing around 40 ml/kg/day due to higher tendency of polyuria compared with most other brain-injured patients. Triggers for application of haemodynamic monitoring with TPT have been defined in the algorithm, including subsequent haemodynamic goals and ‘stopping rules’. Because the target organ concerns the brain, consciousness assessed with the Glasgow Coma Scale (GCS) is included in the algorithm assuming that a perfectly awake patient will constitute a patient with adequate CBF. The protocol is usually adhered to for up to 5 days. Related co-morbidities and circumstances that are quite frequent in brain-injured patients (diabetes insipidus, cerebral salt wasting, osmotic therapies for increased ICP) are not within the scope of this review and the reader is referred to existing literature [50, 72].

## Epilogue

**Fig. 3:**
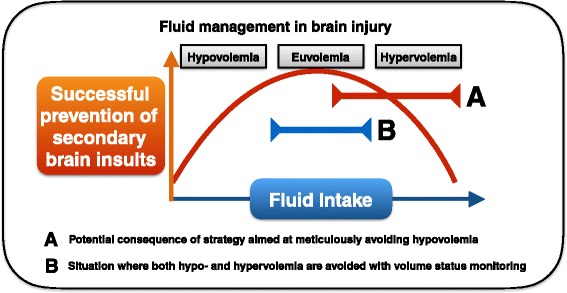
Conceptual explanation of the relation between volume status, fluid intake and risk of secondary brain injury (SBI) in critically ill brain-injured patients. Both hypovolemia and hypervolemia may contribute to SBI. More research is necessary to confirm this concept and establish its clinical significance

The scarce available evidence indicates that fluid management in brain-injured patients should generally be targeted at euvolemia using isotonic fluids. Consequently, it seems that not only ‘too dry’ but also ‘too wet’ is detrimental [62, 69]. Avoiding strong deviations from ‘normality’ therefore seems the best option for most brain-injured patients (Fig. 3). However, routine fluid management is complicated by the circumstance that fluid overload, by definition pertaining to extravascular fluid accumulation in contrast to hypovolemia or euvolemia, is difficult to assess in the brain. This may be an important explanation of why the incidence and potential risks of fluid overload or ‘hypervolemia’ in haemodynamically stable brain-injured patients are understudied in contrast to the emerging literature on this topic in the non-brain-injured critically ill patient [2]. It is important to note that the current literature on fluid management in brain-injured patients has had a main focus on SAH, which is probably related to the well-known risk of hypovolemia associated with cerebral salt wasting syndrome after SAH, whereas studies on fluid management in TBI, ICH and ischaemic stroke are much less numerous. Whether this imbalance in fluid management studies between different types of brain injuries is a reflection of differences in clinical relevance of fluid management is not clear.

It is intriguing that fluid balances seem less clearly associated with secondary brain injuries than fluid intake (especially after SAH). This may indicate that ‘fluid throughput’ may be harmful [1], but it is unknown how exactly this may contribute to brain injury. One may hypothesize that even very small amounts of fluid extravasated to the brain extravascular interstitium may have a significant impact on brain compliance in an already ‘tight’ situation, although such small amounts of fluid extravasates may not be detectable in net fluid balances. Scarce data suggest that normal saline loading, which is a ubiquitous practice in brain-injury management, may have a significant impact on cerebral oedema formation when the BBB is disrupted [73].

Endpoints of fluid management should be clearly defined in future investigations; these endpoints may concern derived parameters of cerebral perfusion and oxygenation when direct effects of (systemic) fluid management on the brain are examined, such as PBrO_2_ [61], or clinical outcome endpoints, such as the modified Rankin scale. In view of the often indirect and complicated relation between fluid management and CBF (Fig. 1), it may be more sensible to focus on associations between cerebral perfusion or function and fluid management in larger populations to uncover potentially deleterious aspects of fluid management, for instance with comparative effectiveness research approaches exploiting the impact of between-centre practice differences to study best practices [74] or prospective randomized studies. When such studies are undertaken, interventions may include haemodynamic monitoring in selected patients deemed at high risk for deviations from euvolemic status. In addition, it is proposed that in such studies details of other medical treatments that may impact on prognosis (and thus confound statistical associations) are meticulously reported, such as blood glucose monitoring and temperature management. We have recently found in high-risk SAH patients that application of a protocolled TPT fluid management protocol, based on fluid responsiveness, resulted in significantly less fluid intake and increased diuresis after starting the protocol (Fig. 2) [75]. The increase in diuresis was accompanied by a significant decrease in CVP (unpublished observation). In our view this may have indicated the presence of venous congestion due to fluid overload prior to the TPT protocol. Increased CVP was related to hypervolemic fluid therapy and more positive fluid balances in several of the referenced investigations in Additional file 1 [27, 46, 47]. These associations, however, should be regarded as contentious and ‘hypothesis generating’ at present.

Although monitoring and treatment aimed directly at the injured brain is an important area of intense research, the data presented seem to indicate that appropriate fluid management is clinically relevant. This notion is in line with previous research indicating that systemic complications and management may have a major impact on mortality in critically ill brain-injured patients [3].

## Conclusion

Routine fluid management may influence clinical outcomes in brain-injured patients. However, the impact of fluid management on brain pathophysiology is complicated due to many intermediate factors governing their relationship. More recent literature has indicated that hypervolemia may be detrimental similar to non-brain-injured critically ill patients. However, research on consequences of fluid overload is seriously hampered by a lack of uniform definitions and the fact that cerebral oedema is difficult to routinely assess. Although the general aim of fluid management in critically ill brain-injured patients is euvolemia using isotonic fluids, ascertainment of euvolemia is problematic in routine clinical practice without haemodynamic monitoring. Therefore, awareness of potential harm from both hypovolemia and hypervolemia may currently be insufficient.

## Additional file

Additional file 1: is a table presenting a summary of salient features of contemporary studies in neurocritical care patients reporting associations of fluid balances or fluid intake with relevant clinical outcomes or haemodynamic variables. (DOCX 80 kb)

## Abbreviations

BBB: blood–brain barrier
CBF: cerebral blood flow
CVP: central venous pressure
CSF: cerebrospinal fluid
DCI: delayed cerebral ischaemia
DIND: delayed ischaemic neurologic deficit
GEDI: global end-diastolic index
ICH: intracranial haemorrhage
ICP: intracranial pressure
PAOP: pulmonary artery occlusion pressure (wedge)
PBrO_2_: partial pressure of brain tissue oxygen
PEEP: positive-end expiratory pressure
SAH: subarachnoid haemorrhage
TBI: traumatic brain injury
TPT: transpulmonary thermodilution
triple-H: hypertensive hypervolemic haemodilution

## References

[1] Orfanakis A, Brambrink AM (2013). Long-term outcome call into question the benefit of positive fluid balance and colloid treatment after aneurysmal subarachnoid hemorrhage. Neurocrit Care.

[2] Acheampong A, Vincent JL (2015). A positive fluid balance is an independent prognostic factor in patients with sepsis. Crit Care..

[3] Mascia L, Sakr Y, Pasero D, Payen D, Reinhart K, Vincent JL (2008). Sepsis Occurrence in Acutely Ill Patients I. Extracranial complications in patients with acute brain injury: a post-hoc analysis of the SOAP study. Intensive Care Med.

[4] Meyer R, Deem S, Yanez ND, Souter M, Lam A, Treggiari MM (2011). Current practices of triple-H prophylaxis and therapy in patients with subarachnoid hemorrhage. Neurocrit Care.

[5] Velly LJ, Bilotta F, Fabregas N, Soehle M, Bruder NJ, Nathanson MH (2015). Anaesthetic and ICU management of aneurysmal subarachnoid haemorrhage: a survey of European practice. Eur J Anaesthesiol.

[6] Diringer MN, Bleck TP, Claude Hemphill J, Menon D, Shutter L, Vespa P (2011). Critical care management of patients following aneurysmal subarachnoid hemorrhage: recommendations from the Neurocritical Care Society's Multidisciplinary Consensus Conference. Neurocrit Care.

[7] Connolly ES, Rabinstein AA, Carhuapoma JR, Derdeyn CP, Dion J, Higashida RT (2012). Guidelines for the management of aneurysmal subarachnoid hemorrhage: a guideline for healthcare professionals from the American Heart Association/American Stroke Association. Stroke.

[8] Stokum JA, Gerzanich V, Simard JM (2016). Molecular pathophysiology of cerebral edema. J Cereb Blood Flow Metab.

[9] Shackford SR, Zhuang J, Schmoker J (1992). Intravenous fluid tonicity: effect on intracranial pressure, cerebral blood flow, and cerebral oxygen delivery in focal brain injury. J Neurosurg.

[10] Tommasino C, Moore S, Todd MM (1988). Cerebral effects of isovolemic hemodilution with crystalloid or colloid solutions. Crit Care Med.

[11] Ertmer C, Van Aken H (2014). Fluid therapy in patients with brain injury: what does physiology tell us?. Crit Care.

[12] Michinaga S, Koyama Y (2015). Pathogenesis of brain edema and investigation into anti-edema drugs. Int J Mol Sci.

[13] Leone M, Asfar P, Radermacher P, Vincent JL, Martin C (2015). Optimizing mean arterial pressure in septic shock: a critical reappraisal of the literature. Crit Care..

[14] Luce JM, Huseby JS, Kirk W, Butler J (1982). A Starling resistor regulates cerebral venous outflow in dogs. J Appl Physiol Respir Environ Exerc Physiol.

[15] Kurishima C, Tsuda M, Shiima Y, Kasai M, Abe S, Ohata J (2011). Coupling of central venous pressure and intracranial pressure in a 6-year-old patient with fontan circulation and intracranial hemorrhage. Ann Thorac Surg.

[16] Mascia L, Grasso S, Fiore T, Bruno F, Berardino M, Ducati A (2005). Cerebro-pulmonary interactions during the application of low levels of positive end-expiratory pressure. Intensive Care Med.

[17] Hariri RJ, Firlick AD, Shepard SR, Cohen DS, Barie PS, Emery JM (1993). Traumatic brain injury, hemorrhagic shock, and fluid resuscitation: effects on intracranial pressure and brain compliance. J Neurosurg.

[18] Trevisani GT, Shackford SR, Zhuang J, Schmoker JD (1994). Brain edema formation after brain injury, shock, and resuscitation: effects of venous and arterial pressure. J Trauma.

[19] Le Roux P, Menon DK, Citerio G, Vespa P, Bader MK, Brophy GM (2014). Consensus summary statement of the International Multidisciplinary Consensus Conference on Multimodality Monitoring in Neurocritical Care: a statement for healthcare professionals from the Neurocritical Care Society and the European Society of Intensive Care Medicine. Intensive Care Med.

[20] Jauch EC, Saver JL, Adams HP, Bruno A, Connors JJ, Demaerschalk BM (2013). Guidelines for the early management of patients with acute ischemic stroke: a guideline for healthcare professionals from the American Heart Association/American Stroke Association. Stroke.

[21] Wijdicks EF, Sheth KN, Carter BS, Greer DM, Kasner SE, Kimberly WT (2014). Recommendations for the management of cerebral and cerebellar infarction with swelling: a statement for healthcare professionals from the American Heart Association/American Stroke Association. Stroke.

[22] Brain Trauma Foundation, American Association of Neurological Surgeons, Congress of Neurological Surgeons (2007). Guidelines for the management of severe traumatic brain injury. J Neurotrauma.

[23] Mutoh T, Kazumata K, Ajiki M, Ushikoshi S, Terasaka S (2007). Goal-directed fluid management by bedside transpulmonary hemodynamic monitoring after subarachnoid hemorrhage. Stroke.

[24] Hoff RG, van Dijk GW, Algra A, Kalkman CJ, Rinkel GJ (2008). Fluid balance and blood volume measurement after aneurysmal subarachnoid hemorrhage. Neurocrit Care.

[25] Mutoh T, Kazumata K, Ishikawa T, Terasaka S (2009). Performance of bedside transpulmonary thermodilution monitoring for goal-directed hemodynamic management after subarachnoid hemorrhage. Stroke.

[26] Hoff RG, Rinkel GJ, Verweij BH, Algra A, Kalkman CJ (2010). Pulmonary edema and blood volume after aneurysmal subarachnoid hemorrhage: a prospective observational study. Crit Care.

[27] Martini RP, Deem S, Brown M, Souter MJ, Yanez ND, Daniel S (2012). The association between fluid balance and outcomes after subarachnoid hemorrhage. Neurocrit Care.

[28] Gura M, Elmaci I, Cerci A, Sagiroglu E, Coskun KK (2012). Haemodynamic augmentation in the treatment of vasospasm in aneurysmal subarachnoid hemorrhage. Turk Neurosurg.

[29] Watanabe A, Tagami T, Yokobori S, Matsumoto G, Igarashi Y, Suzuki G (2012). Global end-diastolic volume is associated with the occurrence of delayed cerebral ischemia and pulmonary edema after subarachnoid hemorrhage. Shock.

[30] Kuwabara K, Fushimi K, Matsuda S, Ishikawa KB, Horiguchi H, Fujimori K (2013). Association of early post-procedure hemodynamic management with the outcomes of subarachnoid hemorrhage patients. J Neurol.

[31] Ibrahim GM, Macdonald RL (2013). The effects of fluid balance and colloid administration on outcomes in patients with aneurysmal subarachnoid hemorrhage: a propensity score-matched analysis. Neurocrit Care.

[32] Tagami T, Kuwamoto K, Watanabe A, Unemoto K, Yokobori S, Matsumoto G (2014). Effect of triple-H prophylaxis on global end-diastolic volume and clinical outcomes in patients with aneurysmal subarachnoid hemorrhage. Neurocrit Care.

[33] Tagami T, Kuwamoto K, Watanabe A, Unemoto K, Yokobori S, Matsumoto G (2014). Optimal range of global end-diastolic volume for fluid management after aneurysmal subarachnoid hemorrhage: a multicenter prospective cohort study. Crit Care Med.

[34] Mutoh T, Kazumata K, Terasaka S, Taki Y, Suzuki A, Ishikawa T (2014). Early intensive versus minimally invasive approach to postoperative hemodynamic management after subarachnoid hemorrhage. Stroke.

[35] Togashi K, Joffe AM, Sekhar L, Kim L, Lam A, Yanez D (2015). Randomized pilot trial of intensive management of blood pressure or volume expansion in subarachnoid hemorrhage (IMPROVES). Neurosurgery.

[36] Joffe AM, Khandelwal N, Hallman MR, Treggiari MM (2015). Assessment of circulating blood volume with fluid administration targeting euvolemia or hypervolemia. Neurocrit Care.

[37] Kissoon NR, Mandrekar JN, Fugate JE, Lanzino G, Wijdicks EF, Rabinstein AA (2015). Positive fluid balance is associated with poor outcomes in subarachnoid hemorrhage. J Stroke Cerebrovasc Dis.

[38] Mutoh T, Kazumata K, Yokoyama Y, Ishikawa T, Taki Y, Terasaka S (2015). Comparison of postoperative volume status and hemodynamics between surgical clipping and endovascular coiling in patients after subarachnoid hemorrhage. J Neurosurg Anesthesiol.

[39] Rodling Wahlstrom M, Olivecrona M, Nystrom F, Koskinen LO, Naredi S (2009). Fluid therapy and the use of albumin in the treatment of severe traumatic brain injury. Acta Anaesthesiol Scand.

[40] Ichai C, Payen JF, Orban JC, Quintard H, Roth H, Legrand R (2013). Half-molar sodium lactate infusion to prevent intracranial hypertensive episodes in severe traumatic brain injured patients: a randomized controlled trial. Intensive Care Med.

[41] Yumoto T, Sato K, Ugawa T, Ichiba S, Ujike Y (2015). Prevalence, risk factors, and short-term consequences of traumatic brain injury-associated hyponatremia. Acta Med Okayama.

[42] Clifton GL, Miller ER, Choi SC, Levin HS (2002). Fluid thresholds and outcome from severe brain injury. Crit Care Med.

[43] Ferrada P, Evans D, Wolfe L, Anand RJ, Vanguri P, Mayglothling J (2014). Findings of a randomized controlled trial using limited transthoracic echocardiogram (LTTE) as a hemodynamic monitoring tool in the trauma bay. J Trauma Acute Care Surg.

[44] Elmer J, Hou P, Wilcox SR, Chang Y, Schreiber H, Okechukwu I (2013). Acute respiratory distress syndrome after spontaneous intracerebral hemorrhage. Crit Care Med.

[45] Fletcher JJ, Bergman K, Blostein PA, Kramer AH (2010). Fluid balance, complications, and brain tissue oxygen tension monitoring following severe traumatic brain injury. Neurocrit Care.

[46] Lennihan L, Mayer SA, Fink ME, Beckford A, Paik MC, Zhang H (2000). Effect of hypervolemic therapy on cerebral blood flow after subarachnoid hemorrhage: a randomized controlled trial. Stroke.

[47] Egge A, Waterloo K, Sjoholm H, Solberg T, Ingebrigtsen T, Romner B (2001). Prophylactic hyperdynamic postoperative fluid therapy after aneurysmal subarachnoid hemorrhage: a clinical, prospective, randomized, controlled study. Neurosurgery.

[48] Schmoker JD, Shackford SR, Wald SL, Pietropaoli JA (1992). An analysis of the relationship between fluid and sodium administration and intracranial pressure after head injury. J Trauma.

[49] Gantner D, Moore EM, Cooper DJ (2014). Intravenous fluids in traumatic brain injury: what's the solution?. Curr Opin Crit Care.

[50] Wright WL (2012). Sodium and fluid management in acute brain injury. Curr Neurol Neurosci Rep.

[51] Tseng MY, Hutchinson PJ, Kirkpatrick PJ (2008). Effects of fluid therapy following aneurysmal subarachnoid haemorrhage: a prospective clinical study. Br J Neurosurg.

[52] Myburgh J, Cooper DJ, Finfer S, Bellomo R, Norton R, SAFE Study Investigators, Australian and New Zealand Intensive Care Society Clinical Trials Group, Australian Red Cross Blood Service, George Institute for International Health (2007). Saline or albumin for fluid resuscitation in patients with traumatic brain injury. N Engl J Med.

[53] Baker AJ, Park E, Hare GM, Liu E, Sikich N, Mazer DC (2008). Effects of resuscitation fluid on neurologic physiology after cerebral trauma and hemorrhage. J Trauma.

[54] Suarez JI, Martin RH, Calvillo E, Dillon C, Bershad EM, Macdonald RL (2012). The Albumin in Subarachnoid Hemorrhage (ALISAH) multicenter pilot clinical trial: safety and neurologic outcomes. Stroke.

[55] Lehmann L, Bendel S, Uehlinger DE, Takala J, Schafer M, Reinert M (2013). Randomized, double-blind trial of the effect of fluid composition on electrolyte, acid-base, and fluid homeostasis in patients early after subarachnoid hemorrhage. Neurocrit Care.

[56] Cooper DJ, Myburgh J, Heritier S, Finfer S, Bellomo R, Billot L (2013). Albumin resuscitation for traumatic brain injury: is intracranial hypertension the cause of increased mortality?. J Neurotrauma.

[57] Gress DR, Participants in the International Multi-Disciplinary Consensus Conference on the Critical Care Management of Subarachnoid Hemmorhage (2011). Monitoring of volume status after subarachnoid hemorrhage. Neurocrit Care.

[58] Taccone FS, Citerio G, Participants in the International Multi-disciplinary Consensus Conference on Multimodality Monitoring (2014). Advanced monitoring of systemic hemodynamics in critically ill patients with acute brain injury. Neurocrit Care.

[59] Maire FW, Patton HD (1956). Role of the splanchnic nerve and the adrenal medulla in the genesis of preoptic pulmonary edema. Am J Physiol.

[60] Hamzaoui O, Georger JF, Monnet X, Ksouri H, Maizel J, Richard C (2010). Early administration of norepinephrine increases cardiac preload and cardiac output in septic patients with life-threatening hypotension. Crit Care.

[61] Kurtz P, Helbok R, Ko SB, Claassen J, Schmidt JM, Fernandez L (2014). Fluid responsiveness and brain tissue oxygen augmentation after subarachnoid hemorrhage. Neurocrit Care.

[62] Muench E, Horn P, Bauhuf C, Roth H, Philipps M, Hermann P (2007). Effects of hypervolemia and hypertension on regional cerebral blood flow, intracranial pressure, and brain tissue oxygenation after subarachnoid hemorrhage. Crit Care Med.

[63] Bouma GJ, Muizelaar JP (1990). Relationship between cardiac output and cerebral blood flow in patients with intact and with impaired autoregulation. J Neurosurg.

[64] Osman D, Ridel C, Ray P, Monnet X, Anguel N, Richard C (2007). Cardiac filling pressures are not appropriate to predict hemodynamic response to volume challenge. Crit Care Med.

[65] Moretti R, Pizzi B (2010). Inferior vena cava distensibility as a predictor of fluid responsiveness in patients with subarachnoid hemorrhage. Neurocrit Care.

[66] Boyd JH, Forbes J, Nakada TA, Walley KR, Russell JA (2011). Fluid resuscitation in septic shock: a positive fluid balance and elevated central venous pressure are associated with increased mortality. Crit Care Med.

[67] Kassell NF, Peerless SJ, Durward QJ, Beck DW, Drake CG, Adams HP (1982). Treatment of ischemic deficits from vasospasm with intravascular volume expansion and induced arterial hypertension. Neurosurgery.

[68] Awad IA, Carter LP, Spetzler RF, Medina M, Williams FC (1987). Clinical vasospasm after subarachnoid hemorrhage: response to hypervolemic hemodilution and arterial hypertension. Stroke.

[69] Dankbaar JW, Slooter AJ, Rinkel GJ, Schaaf IC (2010). Effect of different components of triple-H therapy on cerebral perfusion in patients with aneurysmal subarachnoid haemorrhage: a systematic review. Crit Care.

[70] Cecconi M, De Backer D, Antonelli M, Beale R, Bakker J, Hofer C (2014). Consensus on circulatory shock and hemodynamic monitoring. Task Force of the European Society of Intensive Care Medicine. Intensive Care Med.

[71] Lazaridis C (2012). Advanced hemodynamic monitoring: principles and practice in neurocritical care. Neurocrit Care.

[72] Stocchetti N, Maas AI (2014). Traumatic intracranial hypertension. N Engl J Med.

[73] Chen CH, Toung TJ, Sapirstein A, Bhardwaj A (2006). Effect of duration of osmotherapy on blood-brain barrier disruption and regional cerebral edema after experimental stroke. J Cereb Blood Flow Metab.

[74] Maas AI, Menon DK, Steyerberg EW, Citerio G, Lecky F, Manley GT (2015). Collaborative European NeuroTrauma Effectiveness Research in Traumatic Brain Injury (CENTER-TBI): a prospective longitudinal observational study. Neurosurgery.

[75] Bergmans B, Egal M, Van Bommel J, Bakker J, Van der Jagt M. Effects of cardiac-output guided hemodynamic management on fluid administration after aneurysmal subarachnoid hemorrhage. Crit Care. 2014;18 Suppl:455.

