# Supplementary material for: Fluid management of the neurological patient: a concise review
Source: Crit Care. 2016 May 31;20:126. doi: 10.1186/s13054-016-1309-2 (PMC4886412; doi:10.1186/s13054-016-1309-2)
Supplement: Additional file 1: — is a table presenting a summary of salient features of contemporary studies in neurocritical care patients reporting associations of fluid balances or fluid intake with relevant clinical outcomes or haemodynamic variables. (DOCX 80 kb) [file 13054_2016_1309_MOESM1_ESM.docx]

**Additional File 1**. Summary of salient features of contemporary studies in neurocritical care patients reporting associations of fluid balances or fluid intake with relevant clinical outcomes or hemodynamic variables

| **Author, year** | **n** | **Patients, Design (controls +/-)** | **Intervention** | **HDM studied*** | **Main Outcomes** | *FB/FI*/HDM data and outcome associations* |
| --- | --- | --- | --- | --- | --- | --- |
| Mutoh, 2007[23] | 46 | SAH, Clipped pts, C* (-) | TPT* guided management | PiCCO*, protocol aimed at preventing hypovolemia | Cardiac performance and volume status over time | In poor vs good grade SAH on admission: no difference in net daily fluid balances (-500 to +500 ml) or CVPs but increased intake at day 5 (4,3L), higher early (<48h) CI* (>5.0L/min/m2) and EVLWI* in spite of lower early GEDVI*, higher catecholamines in poor grades which correlated linearly with CI |
| Hoff, 2008[24] | 50 | SAH, Pts admitted within 48h fkuid managed toward normovolemia | - | Blood volume (BV) measurement with pulse dye densitometry (PDD) | Does a FB aim of +0,75L daily maintain normovolemia as defined and measured with PDD? | 22% of pts had BV in the severe hypovolemic range (<50 ml/kg, normal values 60-80 ml/kg). Mean daily FB was +0,57L/d. Daily FI day 0-14 3,3-5,2L/d, mean daily FB +0,3 to +1,1L/d. Odds ratio for DCI when at least one BV<50 ml/kg was measured during first week 1,9. Negative FB not associated with DCI. |
| Mutoh, 2009[25] | 116 | SAH, Clipped pts excluding good grade with minor SAH, RCT (+) | TPT guided protocol vs control: euvolemia based on CVP 5-8mmHg | PiCCO | DIND, FB, FI, cardiovascular complications | TPT group: less FI (maximal 4,9 vs 5,8L/d, no sign. difference FB, less cardiovascular side effects (12 vs 2%), less DIND (32 vs 48%) |
| Hoff, 2010[26] | 102 | SAH, Consecutive pts, P, C (-) | - | Blood volume measurement with pulse dye densitometry | Intravascular volume with vs without pulmonary edema (PE) | PE vs non-PE pts: blood volume in hypovolemic range (but more diuretics) vs euvolemic; PE group had higher FB (+1.6L PE group vs 1.1L)(ns) |
| Martini,  2011[27] | 356 | SAH, Consecutive pts, R, C (-) | - | MAP*, CVP | Comparison of TCD* vasospasm, cerebral infarction on CT or death in pts with positive vs negative three-day cumulative FB | Pts with positive (vs negative) 3-day cum FB: +3,4L vs -1,9L; 3-day FI 16,7L vs 13,5L; lower haematocrit; higher serum sodium and SOFA score; higher CVP (11 vs 9); more blood product use; more TCD vasospasm and higher hospital length-of-stay; mortality and new infarction similar in both groups |
| Gura, 2012[28] | 58 | SAH, Pts with vasospasm after surgery, R and P, C (+) | Before-after study: hypertensive hypervolemic therapy vs hypertensive normovolemic | - | FI, FB, TCD flow velocities, MAP | Hypervolemic group: FI daily of 8,1L versus 5L in normovolemic group. No other differences in outcomes. FB similar (daily +0,3 to 0,5L)  In hypervolemic group 5 (vs 4) infarctions, hypernatremia in 3, pulmonary oedema in 1 patient |
| Watanabe, 2012[29] | 34 | SAH, Consecutive pts with PiCCO, C (-) | - | - | TPT derived parameters’ associations with DCI or PE from 2 days before occurrence | DCI vs no DCI: 7-day cumulative FB 4,0 vs 3,3L (ns), MAP, CVP (between 8-10mmHg), FB (between+0,4-0,7L/d) showed no differences. DCI vs non-DCI showed: CI 3,5 vs 4,8; SVRI* 2524 vs 1794; GEDI 676 vs 872; ELWI and and GEF* did not differ. PE vs no PE: GEDVI higher (947 vs 766) and GEF lower (21% vs 26%); CVP and CI were similar. GEDI predicted fluid responsiveness better than CVP. |
|  |  |  |  |  |  |  |
| Kuwabara, 2013[30] | 5400 | SAH, Population based study, C (-) | - | - | Weight normalized fluid intake (ml/kg/d) other than blood products before and during DCI period (4-14 days after bleed): associations with mortality, conscious deterioration | Odds ratio’s of fluid intake per ml/kg/d: in pre-DCI period for mortality: 1,02 (p<0.001), consciousness deterioration 1,01 (p=0.001), but in DCI period for mortality 0,95 (p<0.001) and for deterioration 0,95 (p<0.001) |
| Ibrahim, 2013[31] | 413 | SAH, Post-hoc propensity-matched analysis on RCT data, R, C (-) | - | - | DIND, delayed infarcts and clinical outcomes in pts who received colloids or not and who had positive FBs or not during DIND risk period (day 3-14 after bleed) | Pts with DIND vs no DIND: 4,7 vs 3.9L/d FI; no difference in FB (-0,13L/d vs -0,07L/d (ns). Only in severe vasospasm net negative FB was associated with DIND (AUC* 0,70), with optimal cut-off of minus 485 ml |
| Tagami, 2014[32] | 178 | SAH, Consecutive pts with PiCCO in perioperative (coil or clip) period, P, C (+) | - | TPT with PiCCO that was used for strictly passive monitoring (no TPT guided management) | DCI, GOS*, FI and MAP and CI/GEDI in pts with vs without physician directed non-protocolized triple-H (not defined) | Triple-H vs control: FI and MAP higher (FI range of 4-4,5L/d vs 3,5L/d but precise numbers not provided in paper) especially after day 3; no differences in CI, GEDI, DCI, GOS |
| Tagami, 2014[33] | 180 | SAH, Consecutive pts with PiCCO in perioperative (coil or clip) period, P, C (+) | - | TPT with PiCCO that was used for strictly passive monitoring (no TPT guided management) | Association of GEDI with DCI and PE and GEDI thresholds for appropriate fluid management | DCI vs no DCI: net total 14-day FB 1,7L vs 1,3L (ns); 1-3 days after bleed GEDI 739 vs 895; 4-7 days after bleed GEDI 783 vs 870; mean positive FB day 1-3 after bleed higher in DCI: 0,7 vs 0,3L.  PE vs no PE: net total 14-day FB 0,9L vs 1,4L (ns). PE incidence with GEDI cut-off 921: >921 44%, <921 18%. |
| Mutoh, 2014[34] | 160 | SAH, Pts with aneurysm occlusion <24h after bleed, RCT (+) | 14 days of TPT guided vs standard management aiming at FB +750-1500 ml or CVP 5-8 mmHg | TPT guided management and CVP and standard data (e.g. MAP) collected | DCI < 21 days, modified Rankin scale (mRS), new infarction on CT or MRI stratified by WFNS on admission | TPT group vs standard management: overall non-significant trends towards less DCI, PE, ICU length of stay and better mRS. In subanalysis of poor grade pts (WFNS IV-V): TPT vs standard showed significantly less DCI, length of ICU stay. In TPT vs standard group: significantly less FI after day 7 (3-4L vs 4-5L estimated, exact numbers not provided), and lower CVP estimated 6 vs 8 (ns); FB consistently (but ns) < 1L/d (TPT) vs >1L/d (after day 4 in standard group) |
| Togashi, 2015[35] | 20 | SAH, Pts with occluded aneurysms<72h after bleed, RCT factorial designed pilot trial (+) | 10 days of normovolemia CVP 4-8, FB<+0,5L/d, 30ml/kg/d FI; hypervolemia CVP 9-15, FB +1-2L/d, FI 60ml/kg/d, factorial combined to normal or augmented MAP | - | mRS at 6 months and cognitive assessments | Hypervolemia vs normovolemia: FB +0,14 vs -0,52 L/d. FI estimated 6,2 vs 4,0L/d, more serious adverse events occurred in hypervolemia group (1 death, 1 DCI, 2 PE) vs 1 PE in normovolemia group (ns) |
| Joffe, 2015[36] | 39 | SAH, Post-hoc analysis on RCT data, R, C (+) | Hypervolemia vs normovolemia | Blood volume measurements with ^131^iodium-labeled albumin injection | Blood volume in hypervolemia vs normovolemia strategy | Hypervolemia vs normovolemia group: FI 81 vs 41 ml/kg/d; blood volume 54 vs 59 ml/kg (ns). Normovolemic range = 60-80ml/kg. |
| Kissoon, 2015[37] | 288 | SAH, Consecutive pts, R, C (-) | - (standard practice aimed at an ‘even’ FB) | - | Outcomes in pts with positive vs negative FB | Poor vs good outcome (mRS 3-6 vs 1-2): net FB +3,5 vs -0,02L; PE 33 vs 15%; DCI 55 vs 29%; net FB per liter Odds Ratio for poor outcome 1,18 |
| Mutoh, 2015[38] | 73 | SAH, Clipped or coiled patients with TPT monitoring, R, C (+) | - | - | TPT derived parameters in clipped vs coiled pts | Clipped vs coiled pts: initial CI 4,2 vs 3,4 L/min/m2); lower GEDI 656 vs 713; higher EVLWI en FI (numbers not provided, estimated 4 vs 3,5L/d at day 7-9); more pts needed fluid challenges for GEDI normalisation (6,3 vs 4,9) |
| Rodling, 2009[39] | 93 | TBI, Pts with GCS* on admission ≤ 8,  C (-) | ICP management according to Lund concept* | CVP, MAP | Organ failures and mortality | Total FI around 11L both in patients who survived and who died from day 1-4 (more albumin in survivors and more packed cells in those who died). Mortality of 14% after 18 months |
| Ichai, 2013[40] | 60 | TBI, Pts with GCS < 9, stabilized after resuscitation, RCT (+) | 48-h infusion of half-molar sodium lactate vs isotonic saline next to BTF* guideline protocol in both groups | - | Episodes with raised ICP, fluid, sodium, chloride balances within the first 48 h | Less ICP episodes in SL group vs saline group (23 vs 53). FB in SL group after 48 h lower than saline group (5 vs 27 ml/kg). No significant outcome differences. |
| Yumuto, 2015[41] | 82 | TBI, Pts with hemorrhage on initial CT and admitted to an ICU, R, C (-) | -  (standard fluid administered was Ringer’s lactate) | - | Prevalence, risk factors and consequences of TBI associated hyponatremia | Hyponatremic (Na<135mEq/L) vs normonatremic pts: higher FI (day 1-3) of 10,6 vs 9,1L; trends towards worse clinical outcome (ns); FBs not given; more skull base fractures in hyponatremic group |
| Clifton, 2002[42] | 392 | TBI, Post-hoc analysis of a RCT (NABIS:H) | - | - | Hypothesis: negative FB are associated with poor outcome and more critically low CPPs* and ICPs | In all patients from the trial combined, those with mean cumulative FB over the first 96h of < -594 ml had 67% poor outcome vs 54% with higher FBs. |
| Ferrada, 2014[43] | 72 | TBI, Sub analysis of RCT on TTE* for preload estimation in trauma pts resuscitation with systolic BP<100, P, RCT, (+) | Fluid resuscitation with or without TTE | - | FI, blood transfusions, mortality | TTE vs non-TTE group: FI 1,04 vs 2,4L; mortality 15% vs 40%; blood transfusions 17 vs 2; |
| Mascia, 2008[3] | 373 | Mixed acute brain injury population (mainly TBI and SAH), Subanalysis SOAP study*, P, C (-) | - | - | Extracranial organ dysfunction and clinical outcomes | Nonsurvivors had significantly higher mean daily fluid balance than survivors (0,1L vs -0,2L) |
| Fletcher, 2010[45] | 41 | Severe traumatic brain injury patients | - | - | Refractory intracranial hypertension and pulmonary edema | More positive fluid balances were associated with pulmonary edema but not with refractory intracranial hypertension |
| Elmer, 2013[44] | 697 | Patients with primary ICH* requiring mechanical ventilation, R, C(-) | - | - | Acute respiratory distress syndrome (ARDS) | ARDS is common after ICH (27%). Higher FB associated with ARDS (HR* 1,14) |

Differences in outcomes or measures are significant unless stated otherwise. ns= non-significant.

The terms euvolemia in this review and normovolemia as mentioned in this table are exchangeable.

***Legend**:

HDM = hemodynamic monitoring, FB = fluid balance, FI = fluid intake, C = observational cohort study, TPT = transpulmonary thermodilution, PiCCO = Pulse Contour Cardiac Output, P = prospective, R = retrospective, CI = cardiac index, EVLWI = extravascular lung water index, GED(V)I = global end-diastolic volume index (ml/m2), CVP = central venous pressure, SGTD = swan-ganz thermodilution catheter, DIND = delayed ischemic neurologic deficit, CO = cardiac output, MAP = mean arterial pressure, TCD = transcranial Doppler, SVRI = systemic vascular resistance index, GEF = global ejection fraction, GOS = Glasgow Outcome Scale, GCS = Glasgow Coma Scale, BTF = Brain Trauma Foundation protocol [22], CPP = cerebral perfusion pressure, TTE = transthoracic echocardiography, SOAP study (=Sepsis Occurrence in Acutely Ill Patients), ICH = intracerebral haemorrhage, HR = hazard ratio

Lund concept = strategy aimed at ICP (<20mmHg) control and CPP (>50mmHg) maintenance by maintaining colloid osmotic pressure and normovolemia with 20-25% albumin, red blood cells but no synthetic colloids and, neutral to slightly negative net fluid balances are maintained and pressors avoided as much as possible. The strategy is aiming for normal blood values and normovolemic status and avoiding stress and sympathetic activity
